# Prevalence of Multidrug-Resistant and Extensively Drug-Resistant Tuberculosis in Patients with Pulmonary Tuberculosis in Zahedan, Southeastern Iran

**Published:** 2012-01-01

**Authors:** M Metanat, B Sharifi-Mood, Sh Shahreki, S H Dawoudi

**Affiliations:** 1 Research Center for Infectious Diseases and Tropical Medicine, Zahedan University of Medical sciences, Zahedan, Iran

**Keywords:** Pulmonary, Tuberculosis, Multidrug-resistant, Extensively drug-resistant

Dear Editor,

Tuberculosis (TB) is responsible for a large portion of morbidity and mortality worldwide. According to WHO report, tuberculosis is responsible for at least 2 million deaths per year, so 90% of these occurring in developing countries.[1][2] Recently, it has been shown that multidrug-resistance (MDR) and extensively drugresistant tuberculosis (XDR-TB) are the most important factors cause death in patients with tuberculosis.[3][4]Drug resistance of M tuberculosis has been implicated in many etiologies such as inadequate and incomplete treatment, host genetic factors and, HIV infection.[5][6][7][8]The long duration of treatment, large amount of anti-TB drugs and their gastrointestinal side effects, are the most important causes of noncompliance in patients with TB.[4] MDR-TB (when resistance to isoniazid and rifampicin appears in combination), increases the numbers of individuals who need treatment with second-line drugs worldwide.[9][10] After developments of MDR-TB, the development of resistance to second line drugs was another problem.[5] XDR-TB is a form of TB caused by bacteria that are resistant to the most effective anti-TB drugs. XDR explained by resistance to both rifampin and isoniazid, as well as to fluoroquinolones plus an injectable agent and Pre-XDR TB isolates are multidrug resistant bacilli which are resistant to either a fluoroquinolone or an injectable agent, but not both.[11] Some believe that XDR-TB strains have emerged from the mismanagement of multidrug-resistant TB and once created, can spread from one person to another.[7][12] TB and drug resistant tuberculosis is also a serious public health problem in Iran as other developing countries. According to the World Health Organization, the estimated incidence rate of TB in Iran is 28 cases per 100,000 population.[2][6] In a report from Southest of Iran,among patients with PTB in Zahedan, where the incidence rate of TB is higher than other regions of Iran,16% of patients with PTB had MDR TB.[9] XDR-TB is higher in patients with MDR-TB and mortality in XDR-TB is higher than MDR-TB.[7] Recent report in Iran showed that 5.3% of patients with PTB had MDR-TB and of these patients, 10.9% had XDR-TB.[7] Since, there were few reports about MDR-TB and no any data about the prevalence of XDR-TB in Zahedan, we decided to evaluate them.

From January 2007 to January 2008, we evaluated all patients with a positive sputum culture who referred to our hospital (Research Center for Infectious Diseases and Tropical Medicine). First, all patients who had clinical signs and symptoms suggestive of PTB were enrolled. These patients were asked to participate in our study. If they accepted, they underwent testing including Ziehl-Neelsen staining and culture. Patients who subsequently were culture-negative for Mycobacterium tuberculosis were withdrawn from the study. Then, we analyzed all culture-positive cases of Mycobacterium tuberculosis infection referred to our TB center. Primary isolation and culture of Mycobacterium isolates, after NaOH-N-acetylcysteine was done in conformity with standard solid-culture procedures.[13]All isolates were identified as M. tuberculosis complex with use of standard biochemical tests, including niacin activity, production of catalase and nitrate reduction, as well as the registration of pigment production and growth rate. Drug susceptibility testing against isoniazid and rifampicin was done by the proportional method on Lo¨wenstein-Jensen media at a concentration of 0.1 and 0.001 mg/mL.[14] Drug-susceptibility testing against second-line drugs (amikacin, kanamycin, capreomycin, ciprofloxacin, levofloxain) was carried out using 2 critical proportions of 0.1 and 0.001. All of these drugs were purchased from glaxo Chemical. Clinical and Epidemiological Information collected from patients with confirmed cases of TB by trained technicians using standard questionnaires. Information obtained on sex, age, previous history of TB, present address, and associated medical data such as chest radiograph findings and presence of HIV infection. Patients also required to have drug-susceptibility test results that showed resistance to isoniazid and rifampin (as MDR-TB), as well as chest radiograph findings and clinical signs and symptoms that were compatible with PTB. Statistical analysis was performed using SPSS, version 11. Data were analyzed by Chi-Square test and p<0.05 was considered significant.

This prospective study was conducted using a total of eighty eight patients (40% male, 60% female) with positive sputum culture for M. tuberculosis complex. The patients aged between 15 and 94 years. 90% of patients were Iranian and 10% wer Afghan. 12.2% of our patients had MDR-TB (3 females; 6 males) and one subject with pre-XDR-TB (resistant to capreomycin) identified. No case had XDR-TB. 8% of culture positive patients had a negative sputum smear and two cases had HIV infection. Ten patients (8.8%) had a past history of treatment for tuberculosis. Among previously treated patients, 44% had MDRTB. Among patients who had received a new diagnosis, 10% had MDR-TB.

Our results showed that 12.2% of our patients had MDR-TB. One patient with pre-XDR-TB (capreomycin resistanse) identified; and there was no case of XDR-TB. Tuberculosis is a serious public health problem in Iran as other developing countries and MDR-TB is a very important and serious health problem among patients with tuberculosis.[9][10] Extensively drug-resistant tuberculosis has also recently emerged as a global health problem, threatening the success of TB control programs in the world.[7][15][16] There are many reports which show MDR and XDR-TB are increasing worldwide. Recent studies in Iran also showed that both MDR and XDR-TB were increasing especially among patients treated within inappropriate regimens or when patients became selectively non-compliant by not taking all of the 3 or 4 drugs.[4][9][10] Masjedi et al. reported results of susceptibility to first-line drugs performed for 1284 Mycobacterium tuberculosis isolates who were referred to the National Research Institute of Tuberculosis and Lung Diseases (Tehran, Iran) for treatment and diagnosis from 2003 to 2005. Subsequently, the strains which were identified as multidrug-resistant M. tuberculosis (113 isolates) were subjected to susceptibility testing for second line drugs. A total of 12 (10.9%) of 113 multidrug-resistant M. tuberculosis strains were XDR M. tuberculosis.[1] Shamimi and his colleagues reported 2% of 548 patients had MDR-TB.[17] Another study in Southeast Iran by Naserpour and colleages showed that 16% of patients with PTB had MDR-TB.[8] There was no any XDR-TB among patients in these two recent studies. A global survey in reference laboratories for TB isolates during 2000-2004 identified XDR-TB in 17 countries and estimated that10% of the sampled multidrug-resistant (MDR) TB strains were XDR.[18] A study in California during 1993-2006 identified 18 XDR-TB cases who had poor outcomes. None of the patients in California with XDRTB had AIDS.[19]

In conclusion, 12.2% of patients had MDR-TB. There was no patient with XDR-TB in our study. We recommend drug-susceptibility testing on the firstline drugs in any patient with tuberculosis in the beginning of treatment.
